# Supplementary material for: Research on low-Impact development layouts in old urban residential communities based on multi-objective optimization
Source: PLoS One. 2025 May 13;20(5):e0317411. doi: 10.1371/journal.pone.0317411 (PMC12074530; doi:10.1371/journal.pone.0317411)
Supplement: Attachment — Submitted filename: Response to Reviewers.docx [file pone.0317411.s002.docx]

## Response to Reviewers

Dear Editors and Reviewers:

Thank you for your letter and for there viewers concerning comments our manuscript entitled “Research on low-impact development layouts in old urban residential communities based on multi-objective optimization”(ID:PONE-D-24-25100).Those comments are all valuable and very helpful for revising and improving our paper, as well as the important guiding significance to our researches. We have studied comments carefully and have made correction which we hope meet with approval.Revised portion are marked in red in the paper and responds are marked in blue in this letter.The main corrections in the paper and the responds to the reviewer's and editor’s comments are as flowing.

Responds to the reviewer's comments:

**Reviewer #1**: 
1. Abstract has to be formatted as per the journal.

Responds:

Our sincere apologies. We have corrected the abstract section.The detailed corrections are as follows (Line16-30)

‘Challenges such as poor drainage systems and limited space for renovation often arise during the sponge city transformation of old urban residential communities. This research focuses on an old residential community in Beijing utilizing the SWMM in combination with the NSGA-II algorithm. A Pareto optimal curve was identified iteratively, with construction costs, runoff reduction effects, and node overload time as the optimization objectives and the maximum area for the low impact development (LID) layout as a constraint. After transformation using LID measures, the runoff reduction rate is directly proportional to the construction cost. However, the rate of runoff reduction increases marginally when the construction cost exceeds 1.4 million yuan. Additionally, the average node overload time decreases as the construction cost and runoff reduction rate increase. Therefore, selecting a transformation scheme requires comprehensive consideration of factors such as budget and land use type in practice .’

2. There are several issues with citations throughout the manuscript.
"Furthermore, the proliferation of impermeable surfaces reduces rainwater infiltration, leading to lower groundwater levels and increased surface runoff2"

"Randall et al. 9 found through simulation that"
Please use the correct style.

Responds:

Our sincere apologies. We have corrected the mistakes and carefully proofread the manuscript.

3. It is very much interesting to see how you linked SWMM to NSGA II in the events of no library files. Can you briefly explain this.

Responds:

Our sincere apologies,we didn't explain clearly in the manuscript.This study adopted the Python language and used the Pyswmm package to call the SWMM model,then used the PyMOO library directly call the NSGA-II algorithm and custom Elementwise Problem.The detailed corrections are as follows (Line339-342 and Line344-347)

‘This study adopted the Python language and used the Pyswmm package to call the SWMM model, integrating the SWMM simulation process into optimization. PyMOO is an open-source Python library that focuses on solving multi-objective optimization problems.It offers a set of flexible and efficient tools to assist researchers and developers in tackling complex issues, directly utilizing the NSGA-II algorithm and supporting custom ElementwiseProblem. Thereby constructing a true multi-objective optimization model for rainwater pipe networks.’

‘Using Python programming software, the inp file from SWMM was accessed, and NSGA-II algorithms were applied for optimization debugging by PyMOO library. the NSGA-II algorithmrun took about 88 to 90 min on an AMD Ryzen9-7900X desktop personal computer with a 4.70 GHz processor,and the length of computational time depends on the performance of the computer processor.After completing 500 iterations, an optimal set of solutions was identified, considering LID construction costs, catchment area runoff reduction rates, and node overload times as the objectives for optimization.’

4. What is the computational time for the 100 iterations?

Responds:

100 iterations take 1005.0377 seconds(16.75 mins)approximately on an AMD Ryzen9-7900X desktop personal computer with a 4.70 GHz processor,and the length of

computational time depends on the performance of the computer processor.The graph below shows the 100 iterations of the calculation process and the time.

5. It would be better to identify few solutions from the Pareto curve and then to discuss the real behaviour of them in depth!

Responds:

Thank you for your constructive comments. We have revised the section to provide more actionable solutions from the Pareto curve. The details as follows(Line400-402 and Line 412-419）

‘For a precipitation event with a recurrence interval of ten years (P = 10),Five optimal solutions (Scheme I to Scheme V) for case were selected for further analysis which schemes were selected based on ascending construction costs. These schemes were then modeled using SWMM software to evaluate changes in catchment area performance, node response, and environmental benefits. The construction costs for these five LID schemes ranged from 700,000 Yuan to 1,770,000 Yuan, with runoff reduction rates varying between 6.48% and 11.82% and node overload times decreasing from 57.46 minutes to 55.91 minutes. ‘

‘As the construction costs for the five selected schemes progressively increase, a corresponding steady reduction in node waterlogging duration is observed,and the decline rate was relatively average. Among these schemes, Scheme V achieved the shortest waterlogging duration, with a reduction of 0.1 hours (6 minutes) compared to the control group, and exhibited the lowest number of waterlogging nodes, showing a decrease of 6 nodes relative to the control group. The inclusion of random mutation and recombination in the genetic algorithm results in variations in the coverage area of each LID facility, which, in turn, causes fluctuations in the number of waterlogged nodes. Thus, the effectiveness of a scheme is primarily judged based on the duration of waterlogging.’

6. Please see the following paper too...https://doi.org/10.3390/hydrology2040230

Responds:

We appreciate your important comment,this literature give us a great inspiration, so this article have been cited in the manuscript(Lines72-75)

‘Rathnayake considering the migration behavior of storms in control of urban sewer networks and incorporating on-line storage tanks into the SWMM, then calling the NSGA-II algorithm to optimize, analysis shows that the developed multi-objective optimization control algorithm has been improved under the action of storage tanks.’

**Reviewer #2:**

1.The study focuses on a specific historical urban area and relies on the characteristics of that region, making it unclear whether the findings can be applied to other areas. In particular, there is room for debate as to whether the same results can be obtained in regions with different climate conditions and terrain. Unless experiments are conducted over a broader range and comparisons are made with other cities, the generalizability of the results may be lacking. This issue should be addressed in the manuscript.

Responds:

we recognized this as a limitation and discussed it in the limitation section.The details as follows.(Line495-498)

‘Given that the research area constitutes an old, compact urban residential community, the findings of this study are subject to certain limitations. Further research should be conducted in a larger administrative district,with experiments performed in different areas and comparisons made with results from other cities to validate the generalizability of the findings, and additional factors such as the urban water system layout and wastewater treatment and reuse need to be considered.’

2.Although the potential for construction costs to become very high is indicated, it is unclear whether the balance between cost and effect has been sufficiently discussed. In particular, there seems to be a lack of consideration regarding the applicability in cities or regions with strict economic constraints, as well as the long-term maintenance costs. Please expand on this point.

Responds:

We appreciate your professional review of our article. We have used your comments to revise the manuscript and have attached a point-by-point response to your comments. The construction cost of LID reaches maximum efficiency when the controlled runoff quantity reaches 10% in a ten-year recurrence period. However, the construction cost increases at a high rate when it exceeds 10% in this study.

Optimal schemes with varying priorities can be identified by comparing the construction schemes across different cost ranges and analyzing detailed parameters of the catchment areas. Therefore, this study is also applicable to cities or regions with strict economic constraints.

For the long-term maintenance cost control of LID, we have revised the section to provide more actionable suggestions for practitioners, especially in the case of green roofs, sunken green spaces, permeable pavements, and rainwater tanks. In the future design and construction stage, green roofs and sunken green spaces can be selected with vegetation that is adaptable to the local soil and climate, and has strong resistance to pests and diseases, in order to reduce the maintenance cost of vegetation in the later stage. For rainwater tanks and permeable paving, adopt drainage systems that are easy to clean and maintain, choose corrosion-resistant materials, and establish a detailed maintenance plan to regularly inspect and maintain the equipment and materials.

the detailed corrections are as follows (Line 487-493 and Line504-509)

‘The construction cost of LID reaches maximum efficiency when the controlled runoff quantity reaches 10% in a ten-year recurrence period(P=10). However, the construction cost increases at a high speed when it exceeds 10%. This study is also applicable to cities or regions with strict economic constraints,because optimal schemes with varying priorities can be identified by comparing the construction schemes across different cost ranges and analyzing detailed parameters of the catchment areas. However, the final scheme should be selected based on a comprehensive evaluation of various factors, including land use type, climatic conditions, and policy decisions.’

‘In order to control the long-term maintenance cost of LID, in the future design and construction stage, green roofs and sunken green spaces can be selected with vegetation that is adaptable to the local soil and climate, and has strong resistance to pests and diseases, to reduce the maintenance cost of vegetation in the later stage. For rainwater tanks and permeable paving, adopt drainage systems that are easy to clean and maintain, choose corrosion-resistant materials, and establish a detailed maintenance plan to regularly inspect and maintain the equipment and materials.’

3.The data and simulation results used are based on specific weather conditions and models, but it is uncertain whether they can be generalized to all conditions. In particular, results that depend on simulations may not necessarily match real-world applications. Please provide additional details addressing these concerns.

Responds:

It could certainly have an effect.The assumptions and parameter settings used in the simulations may deviate from reality, especially under extreme weather conditions. To address this limitation, we suggest that future research validate the reliability and accuracy of these simulation results under different environmental conditions through field experiments or long-term monitoring data. While simulations offer valuable insights for research, there are still more intricate factors that need to be taken into account in practical applications to enhance the model's applicability.According to your nice suggestions, we have made extensive corrections to our previous draft, the detailed corrections are as follows (Line498-503)

‘The assumptions and parameter settings used in the simulations may deviate from reality, especially under extreme weather conditions. To address this limitation, we suggest that future research validate the reliability and accuracy of these simulation results under different environmental conditions through field experiments or long-term monitoring data. While simulations offer valuable insights for research, there are still more intricate factors that need to be taken into account in practical applications to enhance the model's applicability. ‘

Responds to Journal Requirements:

1. Please ensure that your manuscript meets PLOS ONE's style requirements, including those for file naming. The PLOS ONE style templates can be found at
   https://journals.plos.org/plosone/s/file?id=wjVg/PLOSOne_formatting_sample_main_body.pdf and 
   <https://journals.plos.org/plosone/s/file?id=ba62/PLOSOne_formatting_sample_title_authors_affiliations.pdf>

Responds:

According to your requirements, we have made extensive corrections to our previous manuscript.

2. Please note that PLOS ONE has specific guidelines on code sharing for submissions in which author-generated code underpins the findings in the manuscript. In these cases, all author-generated code must be made available without restrictions upon publication of the work. Please review our guidelines at https://journals.plos.org/plosone/s/materials-and-software-sharing#loc-sharing-code and ensure that your code is shared in a way that follows best practice and facilitates reproducibility and reuse

Responds:

According to your requirements, we have uploaded the code entitled ‘SWMM_nsga2.py’ as other files.

3. Thank you for stating the following financial disclosure: 
"This research was funded by National Natural Science Foundation of China (grant number 42101375) and The APC was funded by Fuqin Liu"

Please state what role the funders took in the study.  If the funders had no role, please state: ""The funders had no role in study design, data collection and analysis, decision to publish, or preparation of the manuscript."" 
If this statement is not correct you must amend it as needed. 
Please include this amended Role of Funder statement in your cover letter; we will change the online submission form on your behalf.

Responds:

According to your requirements,this research was funded by National Natural Science Foundation of China (grant number 42101375) and The APC was funded by Fuqin Liu,and the funder(National Natural Science Foundation of China) had no role in study design, data collection and analysis, decision to publish, or preparation of the manuscript,Fuqin Liu had role in decision to publish.This information has been updated in cover letter.

4. Thank you for stating the following in your Competing Interests section:  
"I have read the journal's policy and the authors of this manuscript have the following competing interest"

Please complete your Competing Interests on the online submission form to state any Competing Interests. If you have no competing interests, please state ""The authors have declared that no competing interests exist."", as detailed online in our guide for authors at http://journals.plos.org/plosone/s/submit-now 
 This information should be included in your cover letter; we will change the online submission form on your behalf.

Responds:

According to your requirements, the authors have declared that no competing interests exist and this information has been updated in cover letter.

5. We note that you have indicated that there are restrictions to data sharing for this study. PLOS only allows data to be available upon request if there are legal or ethical restrictions on sharing data publicly. For more information on unacceptable data access restrictions, please see http://journals.plos.org/plosone/s/data-availability#loc-unacceptable-data-access-restrictions.

Before we proceed with your manuscript, please address the following prompts:

a) If there are ethical or legal restrictions on sharing a de-identified data set, please explain them in detail (e.g., data contain potentially identifying or sensitive patient information, data are owned by a third-party organization, etc.) and who has imposed them (e.g., a Research Ethics Committee or Institutional Review Board, etc.). Please also provide contact information for a data access committee, ethics committee, or other institutional body to which data requests may be sent.

b) If there are no restrictions, please upload the minimal anonymized data set necessary to replicate your study findings to a stable, public repository and provide us with the relevant URLs, DOIs, or accession numbers. For a list of recommended repositories, please see
https://journals.plos.org/plosone/s/recommended-repositories. You also have the option of uploading the data as Supporting Information files, but we would recommend depositing data directly to a data repository if possible.

We will update your Data Availability statement on your behalf to reflect the information you provide.

Responds:

According to your requirements, we have uploaded the data as Supporting Information files.

6. When completing the data availability statement of the submission form, you indicated that you will make your data available on acceptance. We strongly recommend all authors decide on a data sharing plan before acceptance, as the process can be lengthy and hold up publication timelines. Please note that, though access restrictions are acceptable now, your entire data will need to be made freely accessible if your manuscript is accepted for publication. This policy applies to all data except where public deposition would breach compliance with the protocol approved by your research ethics board. If you are unable to adhere to our open data policy, please kindly revise your statement to explain your reasoning and we will seek the editor's input on an exemption. Please be assured that, once you have provided your new statement, the assessment of your exemption will not hold up the peer review process.

Responds:

According to your requirements, the authors have declared that no competing interests exist and this information has been updated in cover letter.

7. We note that Figure 1 in your submission contain map/satellite image which may be copyrighted. All PLOS content is published under the Creative Commons Attribution License (CC BY 4.0), which means that the manuscript, images, and Supporting Information files will be freely available online, and any third party is permitted to access, download, copy, distribute, and use these materials in any way, even commercially, with proper attribution. For these reasons, we cannot publish previously copyrighted maps or satellite images created using proprietary data, such as Google software (Google Maps, Street View, and Earth). For more information, see our copyright guidelines: http://journals.plos.org/plosone/s/licenses-and-copyright.
    
   We require you to either (1) present written permission from the copyright holder to publish these figures specifically under the CC BY 4.0 license, or (2) remove the figures from your submission:
    
   a. You may seek permission from the original copyright holder of Figure 1 to publish the content specifically under the CC BY 4.0 license.  
   We recommend that you contact the original copyright holder with the Content Permission Form (http://journals.plos.org/plosone/s/file?id=7c09/content-permission-form.pdf) and the following text:
   “I request permission for the open-access journal PLOS ONE to publish XXX under the Creative Commons Attribution License (CCAL) CC BY 4.0 (http://creativecommons.org/licenses/by/4.0/). Please be aware that this license allows unrestricted use and distribution, even commercially, by third parties. Please reply and provide explicit written permission to publish XXX under a CC BY license and complete the attached form.”
    
   Please upload the completed Content Permission Form or other proof of granted permissions as an ""Other"" file with your submission.
    
   In the figure caption of the copyrighted figure, please include the following text: “Reprinted from [ref] under a CC BY license, with permission from [name of publisher], original copyright [original copyright year].”
    
   b. If you are unable to obtain permission from the original copyright holder to publish these figures under the CC BY 4.0 license or if the copyright holder’s requirements are incompatible with the CC BY 4.0 license, please either i) remove the figure or ii) supply a replacement figure that complies with the CC BY 4.0 license. Please check copyright information on all replacement figures and update the figure caption with source information. If applicable, please specify in the figure caption text when a figure is similar but not identical to the original image and is therefore for illustrative purposes only.
   The following resources for replacing copyrighted map figures may be helpful:
    
   USGS National Map Viewer (public domain): http://viewer.nationalmap.gov/viewer/
   The Gateway to Astronaut Photography of Earth (public domain): http://eol.jsc.nasa.gov/sseop/clickmap/
   Maps at the CIA (public domain): https://www.cia.gov/library/publications/the-world-factbook/index.html and https://www.cia.gov/library/publications/cia-maps-publications/index.html
   NASA Earth Observatory (public domain): http://earthobservatory.nasa.gov/
   Landsat: http://landsat.visibleearth.nasa.gov/
   USGS EROS (Earth Resources Observatory and Science (EROS) Center) (public domain): http://eros.usgs.gov/#
   Natural Earth (public domain): <http://www.naturalearthdata.com/>

Responds:

According to your requirements, we have removed the figure 1 which contain map/satellite image .

8. Please include captions for your Supporting Information files at the end of your manuscript, and update any in-text citations to match accordingly. Please see our Supporting Information guidelines for more information: http://journals.plos.org/plosone/s/supporting-information.

Responds:

According to your requirements, we have added captions for Supporting Information files at the end of manuscript and updated in-text citations to match accordingly.

We tried our best to improve the manuscript and made some changes in the manuscript.These changes will not influence the content and framework of the paper. And here we did not list the changes but marked in red in revised paper.

We appreciate for Editors/Reviewers’ warm work earnestly, and hope that the correction will meet with approval.

Once again, thank you very much for your comments and suggestions.
